# Evaluating the Environmental Performance and Operational Efficiency of Container Ports: An Application to the Maritime Silk Road

**DOI:** 10.3390/ijerph16122226

**Published:** 2019-06-24

**Authors:** Gang Dong, Jing Zhu, Jin Li, Handong Wang, Yuvraj Gajpal

**Affiliations:** 1 School of Economics and Management, Shanghai Maritime University, Shanghai 201306, China; gangdong@shmtu.edu.cn (G.D.); zydcavalier@163.com (H.W.); 2 School of Business Administration, Southwestern University of Finance and Economics, Chengdu 611130, China; zhuj@swufe.edu.cn; 3 School of Management and E-Business, Key Research Institute-Modern Business Research Center, Zhejiang Gongshang University, Hangzhou 310018, China; 4 Asper School of Business, University of Manitoba, Winnipeg, MB R3T 5V4, Canada; Yuvraj.Gajpal@umanitoba.ca

**Keywords:** container port, environmental performance, operational efficiency, SBM-DEA, MSR

## Abstract

A major goal for port authorities, operators, and investors is to achieve efficient operations and effective environmental protection. This is because the environmental performance of a container port is important for its competitiveness and sustainable development. However, the container ports along the Maritime Silk Road (MSR) have caused numerous problems with the rapid development, among which the most significant problem is environmental pollution. In this paper, we aim to measure and compare the environmental performance and operational efficiency of ten major container ports along the MSR, including the ports of Shanghai, Hong Kong, Singapore, Kelang, Laem Chabang, Colombo, Dubai, Barcelona, Antwerp, and Hamburg. We develop an improved, inseparable data envelopment analysis (DEA) model with slack-based measures (SBMs) to evaluate and compare the environmental performance and operational efficiency, and we incorporate the desirable output of container throughput as well as the undesirable output of CO_2_ emission. Our results show that. Overall. these container ports perform better in terms of operational efficiency than environmental performance. We also provide insights for management and policy makers for container ports with different levels of operational efficiency and environmental performance.

## 1. Introduction

Container ports are key players in international trade and global logistics, and they are considered as critical nodes in maritime supply chains. As more than 80% of global merchandise trade in volume is handled by container ports worldwide, nearly two thirds are located in developing countries. The strategic importance of well-functioning and efficient ports for global economic growth cannot be overemphasized. In particular, global port cargo throughput expanded rapidly in 2017, following two years of weak performance. The top 20 global container ports handled 9.3 billion tons of cargo, which increased from 8.9 billion tons in 2016 [1].

Container ports are principal infrastructural assets that serve shipping and trade, and their performance is largely determined by developments in world economy and trade. As such, international trade, the global supply chain, and the integration among different countries’ economies are heavily dependent on efficient container ports and their associated supply chains. However, today’s landscape of container ports is characterized by fierce competition, especially in the container market segment. Container ports and their stakeholders, more than ever, need to re-evaluate their roles in global maritime supply chains [2]. In particular, enhancing environmental performance and meeting globally established sustainability benchmarks and objectives, such as the Sustainable Development Goals, are increasingly recognized as critical for port planning, investment, and strategic positioning. Therefore, it is important to measure and evaluate the operational and economic performance as well as the social and environmental performances of container ports.

In 2017, eight of the top ten container ports were located in Asia, especially China. Except for Tianjin Port, all ports in China on the top ten list recorded shipping volume increases. The port of Ningbo-Zhoushan ranked first, with the total volume handled surpassing one billion tons for the first time, which rose by 9.5% over 2016. As one of the starting ports of the ancient Maritime Silk Road (MSR), the port of Ningbo-Zhoushan, known as the “living fossil”, was officially launched with this new name on 1 January 2006. Until then, there was not much left on the deep-water shoreline of Ningbo with the rapid growth of port transportation production and the increasing strength of wharf construction. However, Zhoushan, which lies across the sea, was in a “hungry” state for a long time because of the hinterland economy and capital management with abundant deep-water coastline resources to be developed. Under this background, the Ningbo-Zhoushan Port Management Committee was established on 20 December 2005. The port of Ningbo-Zhoushan completed 577 million tons of cargo throughputs in 2009, ranking first in global ports for the first time. In 2017, the total trade volume between Ningbo-Zhoushan and the countries along the MSR was 29.3 billion U.S. dollars. Currently, Ningbo-Zhoushan is not only the world’s largest port but also an international hub port along the MSR [3].

On the other hand, the decreased transportation volume of Tianjin Port reflects the delayed effect of the industrial accident that occurred in 2015, which involved two explosions in the port’s storage and handling of hazardous materials facilities. This incident led to grave concerns in terms of environmental protection and public health. Moreover, the decreased volume also is due to government restrictions on the use of tracks for the carriage of coal. According to fine particulate matter (PM2.5) source analysis, the contribution of vehicle pollution to Tianjin increased from 16% in 2013 to 22% in 2017. Consequently, Tianjin Port placed the control and prevention of pollution from vehicles and ships in a more important strategic position for environmental protection. Moreover, transit transportation with large-size coal vehicles has become one of the major pollution sources affecting air quality in the Beijing–Tianjin–Hebei region. On March 2017, a joint work program on air pollution prevention and control was issued, which banned the carriage of coal by Tianjin Port as well as all ports around the Bohai Sea from September 2017. As a result, Tianjin Port’s import goods, especially coal, continued to decline substantially [4].

Carbon dioxide emissions from international shipping have increasingly been in the spotlight, in particular as they are not covered under the 1997 Kyoto Protocol in the United Nations Framework Convention on Climate Change. Relevant regulations have been considered under the auspices of the International Maritime Organization (IMO) to reduce emissions from international shipping and related guidelines (United Nations Conference on Trade and Development, that is, UNCTAD, 2011; UNCTAD, 2012). After adoption of the Paris Agreement in 2015, further progress has been made to reduce greenhouse emissions from ships. This included the adoption of a road map for developing a comprehensive IMO strategy in 2016 (IMO, 2016, annex 11) and the adoption of an initial strategy in 2018.

In both theory and practice, it is important to address the following important questions in the context of the rapid development of the container ports along the MSR. First, with growing competitive forces affecting the container ports along the MSR, how should we evaluate their operational efficiency? Second, rapid development of container ports along the MSR has also caused various problems, among which the most significant problem is environmental pollution. Therefore, how should we evaluate their environmental performance, including resource conservation and environmental protection? Third, ports have benefited from global economic recovery, which remains still fragile. Facing more challenges arising from the changing dynamics in the liner shipping market, what measures can the ports along the MSR adopt to remain competitive and comply with a heightened global sustainability agenda?

This paper aims to contribute to both academic literature and industrial practices. First of all, we propose a model explicitly considering the undesirable outputs of a container port. Secondly, we adopt a balance index as an objective function under the circumstance of optimal efficiency to improve inseparable input–output slack-based measure (SBM)-DEA. Thirdly, we find the inconsistency between environmental performance and operational efficiency of the container ports along the MSR. Finally, managerial and policy implications are provided to further improve the ten container ports along the MSR.

The rest of this paper is organized as follows. Section 2 gives a literature review on the evaluation of port performance. In Section 3, we propose an improved, inseparable input–output SBM-DEA model. Empirical analyses are performed for the container ports along the MSR in Section 4. Section 5 presents managerial and policy implications by comparing the environmental performance and operational efficiency of these container ports. Conclusions and further potential research directions are presented in Section 6.

## 2. Literature Review

Port performance has received considerable attention in recent years. The related research can be divided into three research streams.

The first research stream evaluates port performance from the perspective of service operations. Panayides and Song [5] defined seaport integration and, accordingly, provided measures in global supply chains. Talley et al. [6] evaluated the effectiveness of a port’s individual services by employing the concept of a port service chain. Wanke and Barros [7] assessed the impacts of public–private partnerships on major Brazilian public ports. They adopted factor extraction of inputs/outputs to compute DEA efficiency estimates. Ding et al. [8] used data envelopment analysis (DEA) and a Malmquist productivity index (MPI) to evaluate operational and productivity changes in 21 small- and medium-sized container port terminals in China. Suárez-Alemán et al. [9] analyzed the performances of container ports in developing countries. They focused on the evolution and drivers of productivity and efficiency. Merkel and Holmgren [10] regressed the estimates on port and country characteristics through a compounded dataset of port efficiency. Kutin et al. [11] analyzed the relative efficiencies of 50 Association of Southeast Asian Nations (ASEAN) container ports and terminals. They applied a traditional output-oriented data envelopment analysis. Schøyen et al. [12] analyzed country-specific measurements from the perspective of port and logistics service delivery outcomes. Ha et al. [13] proposed a measurement instrument for port performance from the perspectives of different stakeholders. They modeled the interdependencies among port performance measures and the combination of weights of interdependent measures.

The second research stream is to measure port performance from the perspective of governance. Zheng and Negenborn [14] compared the two regulation modes of centralization and decentralization. They showed that tariffs, port efficiency, port service demand, and social welfare were higher under a decentralization mode. Zhuang et al. [15] used two duopoly games, a Stackelberg game and a simultaneous game, to model port competition where ports provided differentiated services in the sectors of containerized cargo and dry-bulk cargo. Song et al. [16] investigated motivations for the ports of Flanders (Antwerp, Zeebrugge, Ghent, and Ostend) to choose the strategy of coopetition, the combination of competition and cooperation, to respond to the rapidly changing market environment. Notteboom and Yang [17] illustrated governance factors that have triggered a number of strategic and managerial implications on Chinese ports. Estrada et al. [18] presented a new The Ports Efficiency Performance model (PEP) to study how port cargo openness, productivity level, cargo expansion, and technological adaptability could affect the marginal productivity growth rate and performance of a port. Kang and Kim [19] provided useful insights to assist ports to incorporate sustainability practices in their operations. Their proposed a five-factor model that offered both descriptive and diagnostic tools for the future improvement of port operations. Zhang et al. [20] aimed to answer two basic questions of port governance—namely, how to govern and for what purpose—based on a total sample of 118 studies.

The third research stream is to examine port environmental performances. Bray et al. [21] explored a fuzzy theory based DEA model to assess efficiency for transportation systems considering uncertainty in data. Oliveira and Cariou [22] examined how the degree of competition measured at different levels (local, regional, and global levels) could impact the efficiency score of a given container port. They conducted truncated regression with a parametric bootstrapping model. Na et al. [23] adopted an extended DEA model to estimate the environmental efficiencies of eight container ports in China during 2005–2014. Sun et al. [24] proposed a nonradial DEA preference model, and they found the average efficiency to be low for all ports when environmental factors were considered. Ha et al. [25] developed a new port performance measurement model based on the perspectives from different port stakeholders. They modeled interdependencies among port performance measures and the combination of weights of interdependent measures, with both qualitative and quantitative evaluations of the measures, from multiple stakeholders. Tovar and Wall [26] measured the productivity of Spanish port authorities and identified the drivers of productivity while considering heterogeneity. Lam and Yap [27] conducted a stakeholder analysis for the sustainable development of port cities, emphasizing a sustainable balance among economic, social, and environmental performances. Li et al. [28] used listed enterprises in China’s heavy pollution industry from 2009–2013. They tested the relationship between marketization degree, carbon information disclosure, and the cost of equity financing. Longley et al. [29] monitored multiple pollutants (fine particulate matter (PM2.5), black carbon (BC), elemental composition, and organic diesel tracers, and so on) during autumn 2014. They captured spatial variations in traffic, diesel, and proximity to the port of Auckland.

## 3. Methodological Approach

Data envelopment analysis (DEA) has been a classic performance evaluation methodology in situations where multiple inputs (e.g., the number of employees) and outputs (e.g., the number of products) need be converted into a combined efficiency score [30].

**Figure 1:**
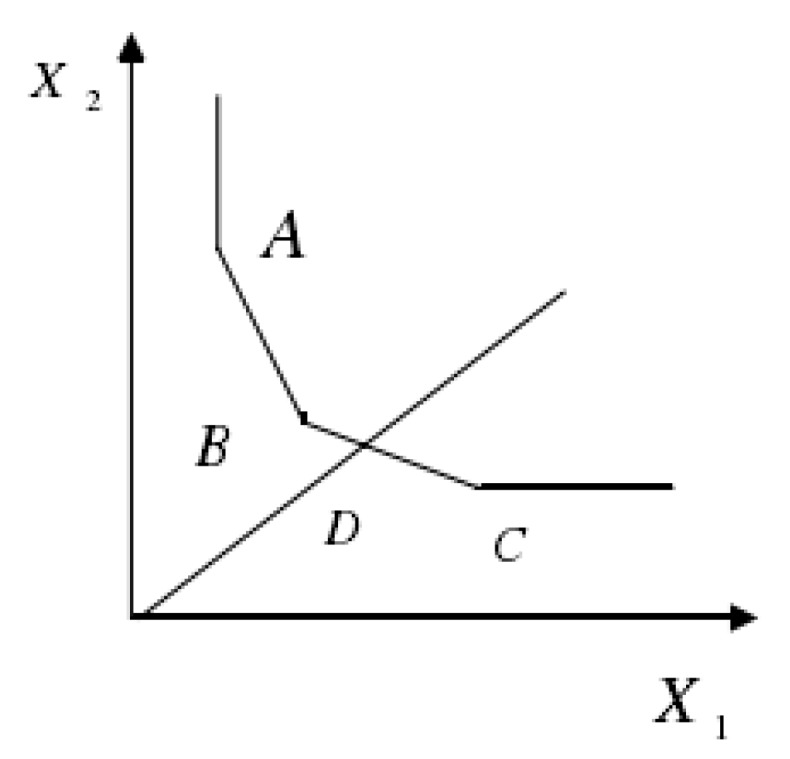
The conceptual graph related to data envelopment analysis (DEA).

The conceptual graph related to DEA is illustrated in Figure 1.

As shown in Figure 1, it is assumed that a production activity needs two kinds of resources, input *X*_1_ and *X*_2_, as well as a corresponding output. Here, there are five decision-making units. The broken line represents the combination of a series of equal output lines composed of input–output piecewise functions. From the graph, it can be seen that decision-making units A, B, C, and D are effective, while other decision-making units are ineffective.

The first DEA model was created by Charnes et al. [31] to analyze the efficiencies of 20 virtual ports in [32]. Subsequently, a number of studies have been done measuring the efficiency of container ports using DEA models. One of them is the CCR-DEA model, which was proposed by A. Charnes, W.W. Cooper, and E. Rhodes [31] in their paper on measuring the efficiency of decision-making units. The CCR-DEA model can be stated as follows:

(1) $$\mathrm{Max}a=\frac{\sum_{j=1}^{N}u_{j}y_{jk}}{\sum_{i=1}^{M}v_{i}x_{ik}}$$

$$s.t.\;\frac{\sum_{j=1}^{N}u_{j}y_{jk}}{\sum_{i=1}^{M}v_{i}x_{ik}}\leq 1$$

$$u_{j},v_{i}\geq 0$$

where *k* denotes the *k*th decision-making unit (DMU), $x_{ik}\left(i=1,2,\ldots ,M\right)\;$and $y_{jk}\left(j=1,2,\ldots ,N\right)$ express the inputs and outputs of the *k*th container port, and $v_{i}\left(i=1,2,\ldots ,M\right)$ and $u_{j}\left(j=1,2,\ldots ,N\right)$ are the weight vectors of the container port’s inputs and outputs.

As a more comprehensive model incorporating the undesirable outputs of a container port, the slack-based measure (SBM) model was proposed by Tone [33] and refined by Lozano and Gutiérrez [34]. This model can simultaneously deal with input reduction and output expansion without sticking to a proportionate change in input and output. For example, Kang et al. [35] adopted the SBM model to measure the environmental efficiency of Taiwanese bus transit firms from 2007–2011, showing that technical efficiency was affected by environmental pollution constraints. Model calibration is mainly based on the research work of Na et al. [23].

Hence, in this study, we measured the environmental performance of container ports along the MSR using the following SBM-DEA model:

$$\mathrm{Min}\beta =\frac{1-\frac{1}{M}\sum_{i=1}^{M}\frac{s_{i}^{-}}{x_{ik}}}{1+\frac{1}{N+Q}\left(\sum_{j=1}^{N}\frac{s_{j}^{d}}{y_{jk}}+\sum_{r=1}^{Q}\frac{s_{r}^{u}}{y_{rk}}\right)}$$

(2) $$s.t.\;\;\;x_{ik}=\sum_{k=1}^{K}\lambda_{k}x_{ik}+s_{i}^{-}$$

$$y_{jk}=\sum_{k=1}^{K}\lambda_{k}y_{jk}-s_{j}^{d}$$

$$y_{rk}=\sum_{k=1}^{K}\lambda_{k}y_{rk}+s_{r}^{u}$$

$$\lambda_{k},s_{i}^{-},s_{j}^{d},s_{r}^{u}\geq 0$$

where $x_{ik}(i=1,2,\ldots ,M$), $\;y_{jk}\left(j=1,2,\ldots ,N\right)$, and $y_{rk}\left(r=1,2,\ldots ,Q\right)$ represent the inputs, the desirable outputs, and the undesirable outputs of the *k*th container port, respectively. $\sum_{k=1}^{K}\lambda_{k}x_{ik}$ and $\sum_{k=1}^{K}\lambda_{k}y_{jk}$ as well as $\sum_{k=1}^{K}\lambda_{k}y_{rk}$ are the linear combinations of the *i*th input, the *j*th desirable output, and the *r*th undesirable output, respectively. $s_{i}^{-}$, $s_{j}^{d}$, and $s_{r}^{u}$ capture the possible improvements of variables.

Consequently, if the value of CCR-DEA or SBM-DEA is equal to one, then the container port is efficient; otherwise, the container port is inefficient. Moreover, when container port values of CCR-DEA or SBM-DEA are all equal to one, that is, in order to completely rank the DMUs using restrictions in DEA model according to the shadow price proposed in Alirezaee and Afsharian [36], $\sum_{j=1}^{N}u_{j}y_{jk}$ and $\sum_{i=1}^{M}v_{i}x_{ik}$ can be regarded as the total revenue and the total cost of *k*th DMU, respectively. Therefore, the constraint of the *k*th DMU can be expressed as:

(3) $$\sum_{j=1}^{N}u_{j}y_{jk}-\sum_{i=1}^{M}v_{i}x_{ik}\leq 0,\;k=1,\ldots ,K$$

When the shadow price comes from technical efficiency, the profit of the *k*th DMU should be equal to zero, which is called the balance state. At this state, the balance index is given by:

(4) $$E_{k}=\sum_{j=1}^{N}u_{jk}q_{j}-\sum_{i=1}^{M}v_{ik}p_{i}$$

where $q_{j}=\sum_{k=1}^{K}y_{jk}$ denotes the sum of *j*th outputs in DMUs. Accordingly, $p_{i}=\sum_{k=1}^{K}x_{jk}$ denotes the sum of *i*th inputs in DMUs. If the values of efficiency are the same, a smaller balance index corresponds to a more effective DMU. Considering the multiple balance indexes, in this paper we improved the extended DEA-based model. This was done by using the balance index as the objective function under the circumstance of optimal efficiency. As a result, the improved model can be written as:

$$\mathrm{Max}(\sum_{j=1}^{N}u_{jk}q_{j}-\sum_{i=1}^{M}v_{ik}p_{i}$$

$$s.t.\;\;\sum_{j=1}^{N}u_{j}y_{jk}-\sum_{i=1}^{M}v_{i}x_{ik}\leq 0$$

$$\sum_{i=1}^{M}v_{i}x_{ik}=1$$

(5) $$\sum_{j=1}^{N}u_{j}y_{jk}=E_{k}$$

$$u_{j},v_{i}\geq 0,k=1,2,\ldots ,K$$

## 4. Evaluating the Container Ports Along the Maritime Silk Road (MSR)

### 4.1. Background

The 21st century Maritime Silk Road (MSR) is one of the two most important components of the Belt and Road Initiative (BRI). The BRI aims to strengthen cooperation priorities, including policy coordination among the countries in the region, facilitate connectivity and trade, and integrate financial and people-to-people bonds (State Information Center, [37]) in the region. As early as 2000 years ago, the MSR started from China’s southeast coastal regions, traversing a vast expanse of oceans and seas to countries in Southeast Asia, Africa, and Europe. The MSR enhanced the exchange of commodities, people, and cultures among the countries situated along the MSR. Even to this day, the MSR continues to be an essential intercontinental transport logistics chain. Taking the largest liner shipping company as example, Maersk Line launched the new Daily Maersk service in 2011, departing daily westbound on the Asia–Europe trade lane, which consists of four port calls in Asia (Ningbo, Shanghai, Yantian, and Tanjung Pelepas) and three calls in northern Europe (Felixstowe, Rotterdam, and Bremerhaven). Soon afterwards, Maersk Line expanded its Daily Maersk offering on the Asia–Europe trade so that Thailand and Indonesia were both included, where Laem Chabang and Jakarta were selected as the loading ports.

In 2017, global container port throughput increased by 6%, which was three times the rate of 2016. Increased port activities reflect the recovery of the world economy and the associated trade flows. In particular, Asia plays a central role in global trade and shipping, as shown by the container shipping sector. The Asia–Pacific region accounts for over 42% of the number of ports and 60% of the calls, in which China represents 19% of all calls alone. The second most important player is Europe, which accounts for 28% of world container ports and 21% of port calls.

In line with the trends in port calls, Asia dominates the container-handling business. The continent continues to account for nearly two-thirds of global container port throughput. Approximately 240 million Twentyfoot Equivalent Units (TEUs) were recorded in China, which represents almost half of all port volumes handled in the region. In 2017, Shanghai remained the busiest container port worldwide with 8.3% expansion in container volumes handled, bringing the total container throughput to 40.2 million TEUs. Singapore ranked the second busiest container port, handling 33.7 million TEUs, a 9% increase over 2016. Shenzhen ranked third, handling 25.2 million TEUs, a 5.1% increase over 2016. The volumes handled in European ports increased by 6.6%, reaching nearly 120 million TEUs, accounting for 16% of global container port throughput. Outside Asia, Rotterdam, Antwerp, Los Angeles, and Hamburg are among the top 20 ports. These ports handled larger volumes in 2017. Particularly, Rotterdam saw the largest increase, where cargo throughput expanded by nearly 10% above the 2016 level.

### 4.2. Data Collection

#### 4.2.1. Input Variables

Considering the historic background and current development of the MSR, ten typical container ports were chosen to carry out evaluations of environmental performance and operational efficiency. These ten ports were Shanghai, Hong Kong, Singapore, Kelang, Laem Chabang, Colombo, Dubai, Barcelona, Antwerp, and Hamburg. According to our model, we adopted the number of berths (*n*) and quay cranes (*n*) as well as the berth length (m) as input variables for the container ports along the MSR. The input variables were added based on different sources such as the Review of Maritime Transport of United Nations Conference on Trade and Development [1], Cargo Systems Top 100 Container Ports of Cargo Systems [38], data from Lloyd’s List of Containers of One Hundred Ports [39], and the official websites and annual report of Port of Shanghai. The values of input variables are presented in Table 1.

#### 4.2.2. Desirable Outputs

Throughput in the container port industry is driven mostly by the development of world economy and global demand, including investment growth, production, and consumption. We used the throughput of the container ports along the MSR as the critical desirable output, which is presented in Table 2.

#### 4.2.3. Undesirable Outputs

Environmental problems that can seriously impact the natural environment and social development of human beings have drawn extensive attention from container port authorities along the MSR. For example, close to 70% of maritime emissions are spreading within 400 km of land, resulting in air pollution in coastal areas. According to the Third IMO greenhouse gas (GHG) Study [40], total shipping emissions were approximately 949 million tons of CO_2_ and 972 million tons of CO_2_ equivalent (CO_2_e) for GHGs including CO_2_, CH_4_, and N_2_O in 2012. International shipping emissions were estimated to be 796 million tons of CO_2_ and 816 million tons of CO_2_e, for approximately 2.2% and 2.1% of global CO_2_ and GHG emissions on a CO_2_e basis, respectively.

We adopted the amount of CO_2_ emission of each container port along the MSR as an undesirable output in evaluating container port environmental performances. We also considered both fuel consumption and power consumption, CO_2_ emissions data were obtained from the Guidelines for National Greenhouse Gas Inventories report published by the Intergovernmental Panel on Climate Change (IPCC) in 2006.

The third GHG Study 2014 was published by the IMO (International Maritime Organization) and provides information about GHG emissions. According to this report, international shipping emitted 796 million tons of CO_2_ in 2012, which accounted for approximately 2.2% of the total emission volume for that year. By contrast, in 2007, before the global economic downturn, international shipping emitted 885 million tons of CO_2_, which represented 2.8% of the global emissions of CO_2_ for that year. Without reference to the findings of this third IMO GHG Study 2014, it would be extremely difficult for IMO to demonstrate the steady and ongoing improvement in ships’ energy efficiencies resulting from the global introduction of mandatory technical and operational measures. The midrange forecasted scenarios presented in this third IMO GHG Study 2014 showed that, by 2050, CO_2_ emissions from international shipping could grow by between 50% and 250%, depending on future economic growth and energy developments.

The CO_2_ emission coefficients and total emissions are summarized in Table 3.

### 4.3. Evaluation and Comparison

#### 4.3.1. Operational Efficiency

According to Golany and Roll [40], isotonicity means any increase in input to the DEA decision-making unit should not reduce output quantity. Therefore, to test data isotonicity before applying DEA, we calculated Pearson correlation coefficients (the correlations between input variables and outputs) in this study according to Table 4. 

From Table 4, we can see that the correlation coefficients between input variables and output variables were positive, and there were generally significant differences. Hence, it can be inferred that the variables in this study were reasonable and suitable for the DEA model. Through model transformation, operational efficiencies of the ten container ports along the MSR were calculated by Matlab 7.0. The results are summarized in Table 5.

In Table 5, the second column contains the operational efficiency from the CCR-DEA model. The values equal to 1 for the ports of Shanghai, Singapore, and Antwerp indicated that these DMUs were efficient. Furthermore, according to the CRA_DEA value of Table 4, the container ports of Shanghai, Singapore, and Antwerp were among the top three efficient ports. They were followed by Hong Kong and Kelang with a CRA_DEA value of 0.978. The operational efficiencies of Hong Kong and Kelang are still relatively high because the CRA_DEA value was close to 1. In addition, the container ports of Hamburg and Dubai achieved CCR-DEA scores of 0.848 and 0.735, respectively. The bottom three ports were Colombo, Barcelona, and Laem Chabang in terms of operational efficiency.

Operational efficiencies of the container ports along the MSR are different due to the use of different types of port mechanical equipment. For exaample, Shanghai Zhenhua Heavy Industries (ZPMC) is a major provider of global port mechanical equipment, launching its crane services organization at various locations around the world including Europe, USA, and in the Middle East. Upgrading the performance of portside cranes can significantly increase productivity on larger vessels while reducing the downtime to a minimum.

#### 4.3.2. Environmental Performance

Environmental performances of the container ports along the MSR were calculated using Equation (2), and the values are reported in Table 6.

As can be seen from Table 5 and Table 6, there was a significant gap between the operational efficiency and the environmental performance in the ports of Kelang, Hong Kong, and Colombo, indicating the container ports of Southeast and Southern Asia along the MSR should pay more attention to improving their environmental performance. The inefficiency (the values of CCR-DEA and SBM-DEA were all lower than 1) of the container ports can be attributed to two major sources: container berth and quay crane inefficiency as well as shore power insufficiency.

#### 4.3.3. Comparison

Therefore, comparison of operational efficiency and environmental performance is illustrated in Figure 1.

**Figure 2:**
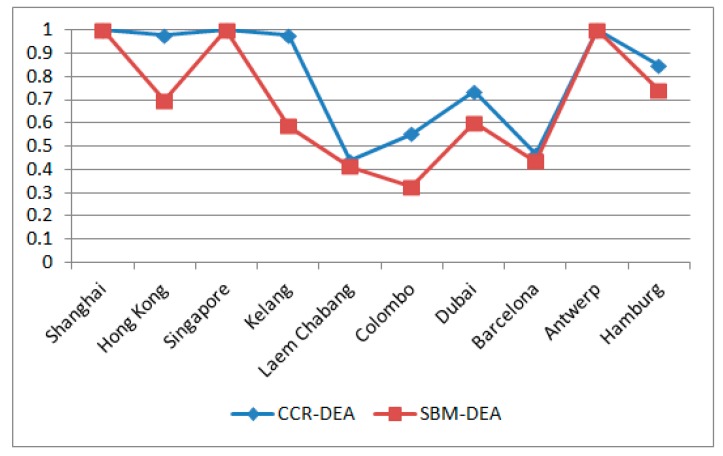
Comparison of the operational efficiency (CCR-DEA) and the environmental performance (SBM-DEA) of the 10 container ports along the MSR.

As shown in Figure 2, the top three container ports along the MSR, in terms of environmental performances, were Singapore, Antwerp, and Shanghai. They were followed by Hamburg, Hong Kong, Dubai, and Kelang. The SBM-DEA scores for all these ports were above 0.5. Therefore, it can be concluded that the environmental performances of these seven ports are relatively high. In contrast, the container ports of Barcelona, Laem Chabang, and Colombo were in the bottom three, with the lowst SBM-DEA value of 0.325 for Colombo. Furthermore, the average operational efficiency and environmental performance of the ten container ports along the MSR were 0.7995 and 0.6797, respectively. Results indicate that the operational efficiency of these ports are better than the environmental performance overall.

The different environmental performances of the container ports along the MSR mainly are due to different port operating systems and design layouts. Fully automated container terminals are becoming the new trend in port industry development. Container ports of Singapore, Shanghai, and Antwerp are all pursuing the direction of automated terminals. For example, a fully automated container terminal can overcome the limitation of a relatively small container yard area, greatly improve utilization of land and deep-water shoreline resources, and further reduce CO_2_ emissions in the processes of container port operation.

#### 4.3.4. Classification

**Figure 3:**
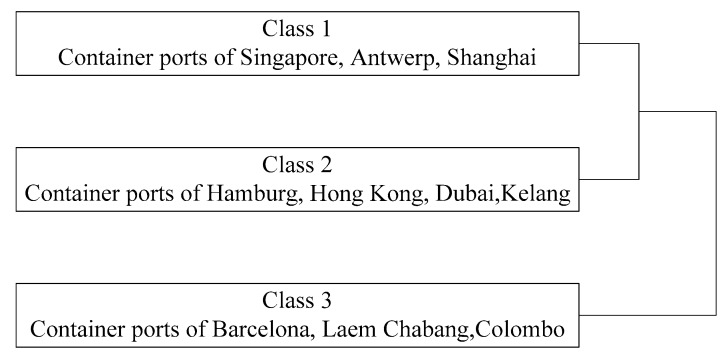
The three classes of the 10 container ports along the MSR.

Based on our evaluation and comparison of the environmental performance and operational efficiency above, the ten container ports along the MSR can be grouped into three classes, as illustrated in Figure 3.

Based on the values of CCR-DEA and SBM-DEA, the ports of Singapore, Antwerp, and Shanghai were categorized into the first class, meaning that they were all efficient. The container ports of Hamburg, Hong Kong, Dubai, and Kelang along the MSR were in the second class, which had a CCR-DEA value of more than 0.7. In particular, the values of Hong Kong and Kelang were 0.978. In addition, the container ports of Hamburg, Hong Kong, Dubai, and Kelang along the MSR were rated relatively low in terms of SBM-DEA, the highest of which was 0.741. In the third class of container ports along the MSR, the ports of Barcelona, Laem Chabang, and Colombo had CCR-DEA and SBM-DEA values around 0.4.

## 5. Managerial and Policy Implications

### 5.1. Infrastructure

As analyzed above, the ports of Hamburg, Hong Kong, Dubai, and Kelang can be categorized as the second class port. For these ports, port management strategies should shift from a cost-saving focus to a sustainability focus by lowering carbon emissions and pollution. To this end, dedicated port infrastructure should be adopted and upgraded. These include constructing shore-based power supply systems for ships; implementing low-voltage, active front end (AFE), four-quadrant frequency conversions for rectifiers and inverters; and achieving a seamless handover in the process of connecting, withdrawing, and changing the ship’s power supply. Moreover, energy generated from loading goods can be integrated into the power grid. Automated guided vehicles (AGVs) and robots equipped with artificial intelligence can be adopted in container port operations. The process of transporting containers from freighter to storehouse involves several steps, each with robots handling a staggering number of sea containers every day without the need for any human intervention.

### 5.2. Management and Policy

The container ports of Singapore, Antwerp, and Shanghai are categorized as the first class port. These ports are opertionally efficient already. Hence, they can focus almost exclusiviely on environemtnal protection to addresss pollution and climate change. This can be done by further reducing greenhouse gas (GHG) emissions, such as CO_2_, NO_2_, and SO_2_ emissions, in their port-related activities. For example, Maritime Singapore Green Initiative seeks to reduce the environmental impact of shipping and related activities and to promote clean and green shipping. For another example, the Ship and Port Pollution Prevention Special Action Plan (2015–2020) was issued in China, which aims to reduce sulfur and nitrogen oxide emissions by up to 65% in major port areas. In October 2018, Shanghai Port implemented an emission control policy. As required by this policy, for all sailing ships, fuel oil with sulfur content less than 0.5% m/m shall be used by international and domestic coastal vessels during their driving and berthing in Shanghai Port.

The third class of container ports along the MSR includes the ports of Barcelona and Laem Chabang as well as Colombo. For them, both environmental performance and the operational efficiency should be simultanesouly improved. As an important international regulatory development adopted by IMO in April 2018, an initial strategy should be implemented to reduce greenhouse gas emissions from ships. A specific goal is to reduce the total annual greenhouse gas emissions from ships by at least 50% by 2050, compared to 2008. Therefore, more attention should be paid on every aspect of the port logistics chain, from coastline planning to general design, construction, operation, and maintenance of container ports.

International shipping is already the most energy-efficient mode for mass transport of cargo. However, the international shipping community must deliver realistic and pragmatic solutions, both from a technical and a political perspective. Under this background, today’s port-operating landscape is characterized by fierce port competition, especially in the container market segment. The decisions made by shipping alliances regarding capacity deployed, ports of call, and network structure can directly affect the performance of a container port terminal. Enhancing port and terminal performance in all market segments is increasingly recognized as a critical component for port planning, investment, and strategic positioning. Efficient terminal performance is also important for meeting globally established sustainability benchmarks such as the Sustainable Development Goals. Ports and their stakeholders, including operators, users, and governments, should collaborate to identify and enable key levers for improving port productivity, profitability, and operational efficiencies.

This study extends previous models by evaluating the environmental performance and operational efficiency of container ports through an improved, inseparable DEA model with slack-based measures. This model can be further extended in several directions. The current model can be further improved by adding the route relationship (i.e., the effect on environmental performance can be analyzed from a transport chain perspective). A multiobjective optimization model can be developed to reflect the combined effects of environmental, commercial, and economic factors. Finally, it would be interesting to include the dynamics of fuel price, fuel sulfur standards, and new energy efficiencies of port equipment and technology in a future study.

## 6. Conclusions and Future Research

The purpose of this paper was to evaluate the operational efficiency and environmental performance of container ports along the MSR. We focused on the major ports of Shanghai, Hong Kong, Singapore, Kelang, Laem Chabang, Colombo, Dubai, Barcelona, Antwerp, and Hamburg. We calculated and compared the values of CCR-DEA and SBM-DEA models of these 10 container ports. Our results show that, overall, these container ports do a better job in terms of operational efficiency than environmental performance. Among these 10 container ports, the ports of Shanghai, Singapore, and Antwerpare were found to be efficient (the values of CCR-DEA and SBM-DEA were all equal to one). The other seven container ports were inefficient (the values of CCR-DEA and SBM-DEA were all lower than one) in terms of operational efficiency and environmental performance. We also provide classification of container ports in three categories, which provides important managerial policies to further improve the performance of ports in MSR.

There are several limitations in this study, which call for future research. First, dynamic adaptability of our model could be pursued by taking uncertainty into account. Second, an empirical analysis should be extended to other container ports along the MSR. Third, considering that port development can be greatly influenced by government policies and regulations, central planning and development of multiple ports can be investigated under emission control and market uncertainty. Last but not least, different environmental measures adopted by the container ports along the MSR can be further analyzed and compared in future research.

## Figures and Tables

**Table 1:** Input variables of the 10 container ports along the Maritime Silk Road (MSR).

Container Port	Country	Number of Container Berths	Container Berth Length (m)	Number of Quay Cranes
Shanghai	China	24	6787	165
Hong Kong	China	19	5950	67
Singapore	Singapore	37	10,300	92
Kelang	Malaysia	18	4900	30
Laem Chabang	Thailand	6	3400	14
Colombo	Sri Lanka	8	2092	18
Dubai	United Arab Emirates	18	4265	82
Barcelona	Spain	5	1440	12
Antwerp	Belgium	36	9210	46
Hamburg	Germany	33	7057	35

Data source: official websites or annual reports of the container ports.

**Table 2:** Throughputs of the 10 container ports along the MSR.

Container Port	Country	2017 (Thousand TEUs)	2016 (Thousand TEUs)	2015 (Thousand TEUs)	2017 (World Rank)
Shanghai	China	40,230	37,135	36,537	1
Hong Kong	China	20,760	19,580	20,114	6
Singapore	Singapore	33,670	30,930	30,962	2
Kelang	Malaysia	12,060	13,167	11,891	12
Laem Chabang	Thailand	7760	7227	6821	20
Colombo	Sri Lanka	6210	5735	5185	24
Dubai	United Arab Emirates	15,440	14,772	15,592	9
Barcelona	Spain	3010	2237	1954	58
Antwerp	Belgium	10,450	10,037	9650	13
Hamburg	Germany	9600	8900	8825	18

Data source: Review of Maritime Transport 2018, One Hundred Ports of Lloyd’s List in 2018; TEUs—Twentyfoot Equivalent Units.

**Table 3:** CO_2_ emissions of the 10 container ports along the MSR.

Container Port	Country	Emission Coefficient (Fuel)	Emission Coefficient (Power)	Emission Amount (Ton)
Shanghai	China	2.150	1.029	86,711.9
Hong Kong	China	1.750	0.839	38,101.5
Singapore	Singapore	0.604	0.731	42,189.6
Kelang	Malaysia	1.103	0.528	32,810.6
Laem Chabang	Thailand	1.218	0.583	27,929.5
Colombo	Sri Lanka	1.527	0.384	21,107.5
Dubai	United Arab Emirates	1.588	0.760	42,140.1
Barcelona	Spain	1.097	0.525	11,592.7
Antwerp	Belgium	0.802	0.289	32,810.6
Hamburg	Germany	1.126	0.539	38,232.4

Data source: Carbon emission coefficient of greenhouse gases of IPCC countries in 2006; Third IMO GHG Study 2014.

**Table 4:** Pearson correlation coefficients between input variables and outputs.

	Number of Container Berths	Container Berth Length	Number of Quay Cranes	Container Throughput	CO_2_ Emission
Number of Container Berths	1.000	-	-	-	-
Container Berth Length	0.958	1.000	-	-	-
Number of Quay Cranes	0.448	0.506	1.000	-	-
Container Throughput	0.494	0.606	0.937	1.000	-
CO_2_ Emission	0.433	0.479	0.940	0.057	1.000

**Table 5:** Operational efficiencies of the 10 container ports along the MSR.

Container Port	CCR-Data Envelopment Analysis (DEA)	Index	Ranking
Shanghai	1.000	−23.154	1
Hong Kong	0.978	−14.802	4
Singapore	1.000	−18.364	2
Kelang	0.978	−9.120	5
Laem Chabang	0.438	−6.780	10
Colombo	0.553	−26.829	8
Dubai	0.735	−17.880	7
Barcelona	0.465	−16.599	9
Antwerp	1.000	−9.367	3
Hamburg	0.848	−10.598	6

**Table 6:** Environmental efficiencies of the 10 container ports along the MSR.

Container Port	Slack-Based Model (SBM)-DEA	Ranking
Shanghai	1.000	3
Hong Kong	0.696	5
Singapore	1.000	1
Kelang	0.587	7
Laem Chabang	0.413	9
Colombo	0.325	10
Dubai	0.601	6
Barcelona	0.434	8
Antwerp	1.000	2
Hamburg	0.741	4
